# The Effect of a Single Dose Oral Pregabalin on Hemodynamic Changes and Duration of Analgesia after Spinal Anesthesia in Orthopedic Surgeries of Tibial Fractures

**Published:** 2018

**Authors:** Alireza Rahat Dahmardeh, Amir Moosavi, Seyed Muhammad Nasir-al-din Tabatabaei, Jamshid Ordoni Avval, Mohammad Sistanizad

**Affiliations:** a *Department of Anesthesiology and Intensive care medicine, Faculty of Medicine, Zahedan Medical University, Zahedan, Iran. *; b *Department of Clinical Pharmacy, Faculty of Pharmacy, Shahid Beheshti University of Medical Sciences, Tehran, Iran. *; c *Department of Pharmaceutical Care Unit, Emam Hossein Medical and Educational Center, Shahid Beheshti University of Medical Sciences, Tehran, Iran.*

**Keywords:** Pregabalin, Hemodynamics, Analgesia, Spinal anesthesia

## Abstract

Treatment of acute pain and hemodynamic changes after surgery is extremely important. Various drugs for pain relief after surgery have been studied. The aim of this study was to evaluate the effect of a single dose of oral pregabalin on hemodynamic changes and duration of analgesia after spinal anesthesia in orthopedic surgeries of tibia fractures. This clinical trial was carried out on 120 patients with fractures of the tibia bone in 2014 in city of Zahedan. Sampling was conducted using randomized blocks and patients were placed into pregabalin (150 mg PO 1 h before anesthesia) and placebo groups. Duration of analgesia, severity of anxiety, mean arterial pressure, and pulse rate were evaluated in both groups. The mean age of the patients was 34.8 ± 21.7 years and 84 patients (70%) were male and 36 (30%) were female. There were no significant differences at baseline characteristics of the patients in two arms of the study. After surgery, mean arterial pressure, pulse rate and anxiety, were significantly lower in intervention group. Also the duration of analgesia was significantly longer in intervention arm (185.3 ± 4.9 vs 36.9 ± 13.9, *P* < 0.01). Dizziness as a side effect of pregabalin was observed in 21 and 11 subjects in intervention and placebo groups, respectively (*P* < 0.01). The present study showed that a single dose of oral pregabalin increases the duration of analgesia and maintains hemodynamic stability in orthopedic surgery patients.

## Introduction

Cardiovascular events after surgery lead to increased complications, decreased physical ability, increased mortality as well as significant increases in health care costs (1).

Increased activity of the sympathetic system increases blood pressure, heart beat, and activity of the clotting factors (2). This increase can cause an imbalance between myocardial oxygen demand and supply. The imbalance can be dangerous to patients with heart problems and intracranial complications (3). Maintaining cardiovascular stability can decrease cardiovascular disease risks caused by increase in the sympathetic nervous system, which appears during surgery (3). Beta-blockers, alpha-two-agonists, clonidine, calcium channel blockers, nitroglycerin, statins, and aspirin can be used to maintain this stability (4, 5).

Pain is one of the most common and annoying problems of patients. Eighty percent of patients undergoing surgical procedures experience postoperative pain. Despite all efforts of pain forums in acute postoperative pain management, this issue is still considered as a serious clinical problem (6, 7). Studies show that pain is an important specification for measuring patient’s satisfaction. Postoperative pain causes various complications in the bodyꞌs organs. Atelectasis, hypoxia, and hypercapnia caused by inadequate ventilation, increased blood pressure and heart rate, myocardial ischemia, cardiac dysrhythmia, hyperglycemia, water and salt retention, weakening of the immune system, increased platelets adhesion and coagulation of constipation and urinary retention are among the most important of these complications. Hence, severity of postoperative pain and method of control are extremely important in prevention and treatment of these complications. Although, opioids are currently the base of pain management after surgery, due to the known side effects of these drugs, there have been many efforts to increase the duration of analgesia. The ultimate goal is to achieve a non-opioid drug with reasonable cost, fewer side effects, and increased duration of action (5, 8).

**Table 1:** Demographic characteristics of the patients in control and pregabalin groups

**Variable**	**Pregabalin (n = 60)**	**Placebo (n** **= 60)**	***P*** **-value**
Age (year)	32.7 ± 13.8	36.9 ± 13.9	0.10
Sex M(%)	44 (63)	40 (57)	0.46

**Table 2:** Comparison of vital signs and anxiety in two arms of the study

**Variable**	**Pregabalin**	**Placebo**	**P-value**
Mean Arterial Pressure (MAP)			
Before	99.7 ± 1.5	97.7 ± 9.2	0.46
After	86.9 ± 3.0	91.5 ± 8.3	0.03
Mean Pulse Rate			
Before	81.3 ± 8.4	81.5 ± 14.2	0.80
After	70.2 ± 8.1	78.4 ± 12.6	0.03
Anxiety (VAS)			
Before	75.7 ± 10.8	71.0 ± 21.4	0.58
After	58.3 ± 11.4	69.7 ± 21.0	0.03

All data have been presented as Mean ± SD.

**Table 3:** Duration of analgesia and adverse effects in two arms of the study

**Variable**	**Pregabalin**	**Placebo**	***P*** **-value**
Duration of analgesia (minute)	185.3 ± 4.9	36.9 ± 13.9	< 0.01
Nausea	23 (38.3%)	19 (31.7%)	0.36
Vomit	22 (36.7%)	18 (30.0%)	0.32
Dizziness	21 (35.0%)	11 (18.3%)	< 0.01

Considering the necessity of pain management in spite of all efforts, there is still no effective method to control pain after surgery and there is need for further research in this area. Searching scientific literature shows that pregabalin has the ability to prevent and control postoperative pain (9).

The best conditions for surgery should be provided in an ideal spinal anesthesia, such as creating necessary anesthesia level and loosening the site of surgery, the time of anesthesia should match the duration of surgery. Moreover, immobility time should not be too long and recovery should be at least in a short possible time. In addition, sufficient analgesia should be maintained for patients in the recovery room after the completion of surgery. 

Therefore, workflow of operating and recovery rooms will be faster and the cost will be lower in the case of achieving these results (10, 11).

Spinal anesthesia is the best type of analgesia in the lower abdomen and lower limb surgery, especially in high-risk patients such as the elderly, obese, and pulmonary heart disease patients who have a higher risk of general anesthesia as compared to the other patients.

Long-acting anesthetic medicines such as tetracaine and marcaine are used in surgical procedures that require a long time (12). Increasing the duration of spinal anesthesia is usually achieved by adding vasoconstrictor medicines such as epinephrine. The use of this vasoconstrictor and their systemic absorption might cause cardiovascular risks and lead to ischemia of spinal cord nerve roots in predisposed patients. Thus, the method that leads to sedentary of patients and increases the duration of analgesia after surgery and do not have the above complications will be an optimal method. Researchers have studied several drugs such as gabapentin and pregabalin in order to achieve this goal (12).

Pregabalin, an analog of gamma amino butyric acid has been designed as a more potent successor of gabapentin and is presented under the brand name Lyrica. It is used for the treatment of neuropathic pains, sleep disorders, anxiety, epilepsy and post-herpetic neuralgia, and diabetic neuropathy. Dizziness and drowsiness among its side effects (more than 10% of patients) and other side-effects (1-10% of patients) are blurred vision, double vision, increased appetite, weight gain, happiness, euphoria, confusion, changes in sexual desire, irritability, lack of balance, shifting attention, paramnesia, increased levels of creatine kinase, etc. (2, 13).

Given that most studies in this field are related to general anesthesia and increase in the duration of analgesia after surgery, and a few studies have been conducted in the field of evaluating the effect of pregabalin on mean arterial pressure, pulse and duration of analgesia after spinal anesthesia, a research was conducted with the aim of determining the effect of a single dose of oral pregabalin on hemodynamic changes and duration of analgesia after spinal anesthesia in orthopedic surgeries of shin bone (tibia) fracture.

## Experimental

In this randomized, double blinded, and placebo control clinical trial, 120 patients were selected from all the patients having tibial fracture and hospitalized in Khatam-Al­-Anbia hospital in 2014 affiliated to Zahedan university of medical sciences, Zahedan, Iran. Inclusion criteria were ASA Class 1 non-emergency patients, aged 20 to 70 years, undergoing tibial fracture orthopedic surgery.

Exclusion criteria were having diseases affecting the hemodynamic situations such as heart disease, diabetes, kidney disease, or a history of allergy to gabapentin or pregabalin, drug use, regular use of alcohol or smoking more than 5 pack year, neurological diseases, surgery duration more than ninety min after spinal anesthesia and patients who needed injections during the operation which affect hemodynamics, pregnancy, refusal of patients to participate in the study and spinal anesthesia, coagulopathy, infection in the area of spinal anesthesia and any digestive disorder that interferes with the absorption of oral medication, the need for paregoric for pain relief, abnormal bleeding during surgery, cardiovascular and cerebrovascular complications during surgery and abnormal pulse and mean arterial pressure.

The study protocol was approved by ethics committee of Zahedan University of Medical Sciences. One hundred and twenty patients were randomly (using the blocked random sampling) divided into the pregabalin group (two 75 mg capsules) or placebo by controlling the dependent variables before and after the intervention. Patients received pregabalin or placebo one hour before spinal anesthesia. Mean arterial pressure, pulse rate and duration of analgesia after spinal anesthesia were recorded for each patient.

For eligible patients, pressure cuff was attached to the left arm ninety minutes before spinal anesthesia and pulse oximeter was connected to the finger of the contralateral limb. Pulse and mean arterial pressure were recorded with the set of each 10 min for 3 times using vital signs monitoring device produced by Sazandegan Gostar Co. Research questionnaire was completed for them when no abnormal symptom was observed. 

Severity of anxiety was recorded before and after surgery using Visual Anxiety Severity (VAS) score. It was asked from the patient with adequate explanations on the method of answering this tool. The used VAS is in the form of a 100 mm horizontal line, which measures the level of anxiety from the lack of it to its most severe level. 

One h before spinal anesthesia, two 75 mg pregabalin capsules (Pfizer, USA) or placebo were taken by the patients based on the randomization Table in the presence of a research assistant. All the patients received 10 mL/Kg Ringer fluid and then spinal anesthesia was performed by 25-gauge Quincke needles from L3-L4 intervertebral space with 3 mL of 0.5% Marcaine. Pain relievers were administered as needed based on the anesthesiologist opinion. The mean arterial pressure and pulse were recorded every 10 min using vital signs monitoring until the end of the surgery. The data were analyzed using relevant statistical tests by SPSS-16 software. 

## Results

One hundred and twenty patients (60 in each group) for orthopedic surgery due to fracture were recruited in this study. The mean age of the patients was 34.8 ± 21.7 years, 84 patients (70%) were male and 36 (30%) were female. There was no significant difference in demographic characteristics of the patients in two arms of the study (Table 1).

There were no significant differences between two groups in mean arterial pressure, pulse rate, and anxiety (measured by VAS) before surgery (Table 2). After receiving pregabalin or placebo the MAP decreased from 99.1 ± 7.5 mm Hg to 88.5 ± 9.5 (*P* = 0.01) in pregabalin arm and from 97.9 ± 7.2 mm Hg to 93.3 ± 6.7 mm Hg in control arm (*P* = 0.12). These reductions for pulse rate in pregabalin and control groups were 81.3 ± 8.4 to 70.2 ± 8.1 beat/min (*P* = 0.001) and 81.5 ± 14.2 to 78.4 ± 12.6 beat/min (*P* = 0.539), respectively. 

The data about duration of analgesia and adverse effects are shown in Table 3. Mean duration of analgesia was 185.3 ± 4.9 min in the experimental group and 129.4 ± 5.6 min in the placebo group. This difference was statistically significant (*P* = 0.001). Frequency of nausea and vomiting was similar between the two groups but the dizziness was significantly higher in the Pregabalin group (*P* = 0.001). This difference is statistically significant (Table 3).

In this study, the average severity of anxiety according to VAS before operation was 75.7 ± 10.8 mm in the Pregabalin group and 71 ± 21.4 mm in the placebo group. This difference was not statistically significant (*P* = 0.585). After operation, it was 58.3 ± 11.4 mm in the Pregabalin group and 69.7 ± 21 mm in the placebo group. This difference was statistically significant (*P* = 0.03) (Table 2).

## Discussion

The results of this study showed that pain was lower in the pregabalin-treated group than the control group. Also, the patientꞌs blood pressure and, pulse rate and VAS were lower in the pregabalin group.

Approximately, 80% of the patients experienced pain after surgery which is known as a predictor of persistent postoperative pain, 5-50% of the patients reported persistent pain. The pain is associated with symptoms such as tachycardia, hypertension, myocardial ischemia, and decreased pulmonary ventilation. It can also increase the susceptibility of neurons and the release of inflammatory mediators.

A study by Kohli and colleagues in 2011 on the use of pregabalin orally one hour before hysterectomy surgery with spinal anesthesia showed that mean arterial pressure and pulse had a significant reduction in the pregabalin group as compared to the placebo (6). In a study in India in 2011, Gupta and colleagues (8) evaluated hemodynamic response to laryngoscopy and laparoscopy and showed that prescription of pregabalin and clonidine before surgery led to hemodynamic stability.

In a study in 2012, Sundar and colleagues showed that a single dose of 150 mg pregabalin undermined blood pressure response to tracheal intubation (9).

In a study by Zhang and colleagues in 2011, a meta-analysis on 45 articles between 2000 and 2010 showed that prescription of single dose of pregabalin before surgery reduced postoperative pain and reduced opioid use (11). The research of Dauri *et al*. (2009) showed that Gabapentin and pregabalin reduced acute pain and opioid use after surgery when compared with placebo (14).

Bafna *et al*. (2014) showed that pregabalin significantly prolongs the analgesia of spinal block as compared to gabapentin (15). In a study which was conducted in 2013 by Eskandar in Egypt, it was determined by prescription of 300 mg of pregabalin in patients undergoing arthroscopic shoulder that Pregabalin increases pain and duration of analgesia after surgery but side effects such as nausea and vomiting were not observed (16).

In a study in 2014 in India, Prasad and colleagues evaluated the duration of analgesia and analgesic drugs use one and half hour before vaginal hysterectomy surgery using anesthesia spinal anesthesia in 90 patients in three groups of placebo, 150 mcg clonidine, and 150 mg oral Pregabalin and showed that prescription pf pregabalin and clonidine before surgery increased the duration of spinal anesthesia and reduced the use of pain medications after surgery when compared with the placebo group (17).

The study of Olmedo and colleagues in 2015 also evaluated the effect of oral Pregabalin in the form of premedication on hemodynamic changes and pain control after surgery of impacted wisdom teeth in which mean changes in systolic were significantly more negative in the intervention group as compared to the control group. Postoperative pain was significantly higher in the control group as compared to the experimental group (18).

Also, in the study of Hassani and colleagues in 2011, analgesic effect of pregabalinin on patients undergoing surgery with gabapentin in different doses were compared and single oral 300 mg dose of pregabalin had more effect in reducing pain than pain killers received within 24 h after Laparoscopy in comparison with 600 and 900 mg of gabapentin (19).

In a study conducted by Alimian *et al*. (2012) with the aim of evaluating the effect of a single dose of oral pregabalin in pain control and reducing the amount of drugs used after minor surgeries, it was shown that taking a single dose of oral pregabalin 150 mg before the minor surgeries can reduce the score of pain and opioid consumption which is consistent with the present study (20).

According to the results of this study and other studies reviewed, it seems that the use of pregabalin reduces pain and the amount of painkillers used after surgery and also leads to hemodynamic stabilization of patients. The present study showed that a single dose of oral pregabalin reduces pain and leads to hemodynamic stabilization of patients. Deterioration of the device can be pointed out as one of the study limitations.

With the results of this study, the use of pregabalin is recommended in orthopedic surgery of patients with spinal anesthesia for reduction of pain and hemodynamic stabilization of patients.
